# Gomisin L1, a Lignan Isolated from Schisandra Berries, Induces Apoptosis by Regulating NADPH Oxidase in Human Ovarian Cancer Cells

**DOI:** 10.3390/life11080858

**Published:** 2021-08-21

**Authors:** Young Hyun Ko, Miran Jeong, Dae Sik Jang, Jung-Hye Choi

**Affiliations:** 1 Division of Molecular Biology, College of Pharmacy, Kyung Hee University, Seoul 02447, Korea; kyh122@khu.ac.kr (Y.H.K.); jeongmr@khu.ac.kr (M.J.); 2 Department of Biomedical and Pharmaceutical Sciences, Kyung Hee University, Seoul 02447, Korea; dsjang@khu.ac.kr

**Keywords:** gomisin L1, Schisandra berry, ovarian cancer, apoptosis, ROS, NOX

## Abstract

The fruits of *Schisandra chinensis* (Schisandra berries) are used as health food supplements and popular food ingredients in East Asia. Lignans, major and characteristic polyphenol compounds of Schisandra berries, possess various biological activities, including hepatoprotective and anticancer effects. However, the biological activities of gomisin L1, a lignan isolated from Schisandra berries, are less to be investigated. In this study, the antitumor activity of gomisin L1 and its underlying molecular mechanism in human ovarian cancer cells were investigated. Gomisin L1 exhibited potent cytotoxic activity against A2780 and SKOV3 ovarian cancer cells. Flow cytometry analysis revealed that the growth inhibitory effects of gomisin L1 were mediated by the induction of apoptosis. Furthermore, gomisin L1 induced an increase in intracellular reactive oxygen species (ROS) levels, and the antioxidant *N*-acetyl cysteine significantly negated gomisin L1-induced cell death. Moreover, inhibition of NADPH oxidase (NOX) using an inhibitor and siRNA attenuated gomisin L1-induced death of, and ROS production in, human ovarian cancer cells. Taken together, these data indicate that the lignan gomisin L1 from Schisandra berries induces apoptotic cell death by regulating intracellular ROS production via NOX.

## 1. Introduction

*Schisandra chinensis* Baillon (Schisandraceae) grows throughout East Asia (Korea, Japan, and China,) and Russian Far East [1]. The fruits of *S. chinensis* (Schisandra berries), known as omiza in Korean, wuweizi in Chinese, and gomishi in Japanese are called five-flavor-fruit and commonly used as health food supplements and food ingredients in East Asia. Schisandra berries have also been used as a traditional medicine to treat coughs, dysentery, spontaneous sweating, and insomnia [2,3]. A phytochemical investigation of Schisandra berries showed the presence of vitamin C, amino acids, citric acid, malic acid, fumaric acid, sesquiterpenes, polysaccharides, tannins, triterpenoids, and lignans [4]. Lignans, the major and characteristic constituents of Schisandraceae plants, have attracted much interest due to their multiple biological and pharmacological activities [5]. Schisandra berries contain over 30 types of lignan, including schizandrin, deoxyschizandrin, wuweizisu C, and gomisins [6,7]. Schisandra fruit extracts and their major bioactive lignans have a wide range of biological activities including anticancer activities [1,4,8,9,10]. However, anti-cancer activities of gomisin L1, a dibenzocyclooctadiene lignan isolated from Schisandra berries, are poorly characterized.

Ovarian cancer has the highest mortality rate of all gynecologic cancers. It is the eighth most common cancer in females worldwide, with an age-standardized incidence ranged from 9.5 to 16.6 per 100,000 females in 2018 [11]. Due to the absence of early symptoms, most patients are diagnosed with advanced-stage disease (stage III or IV) and 5-year survival rates are less than 20%. Most patients are diagnosed with stage III or IV ovarian cancer because clinically effective screening is problematic [12]. The evasion of apoptosis (i.e., programmed cell death) is one of the key characteristics of cancer cells, including ovarian cancer cells. Thus, the anticancer effects of chemotherapeutics such as cisplatin, a first-line chemotherapeutic agent for ovarian cancer, are mainly associated with the induction of apoptosis in cancer cells. In this study, the effects of gomisin L1 on apoptosis and the underlying molecular mechanism in human ovarian cancer cells were investigated.

## 2. Materials and Methods

### 2.1. Materials

The preparation procedure for gomisin L1 has been described previously [13]. Briefly, dried Schisandra berries, *Schisandra chinensis* Baillon fruits, were obtained from Omniherb Co., Ltd. at Kyungdong market (Seoul, Korea), in June 2011. The Schisandra berries were authenticated by Professor Dae Sik Jang and the voucher specimen (no. 2011-SCCH01) has been stored in College of Pharmacy, Kyung Hee University (Seoul, Korea). The air-dried fruits (3.5 kg) were extracted by maceration with 10 L of 80% aqueous ethanol (EtOH) three times. 80% EtOH extract (1.5 kg) was obtained by concentration of the extract in vacuo at 40 °C. The 80% EtOH extract (1.5 kg) was dissolved in deionized water (5 L) and then solvent-partitioned with hexane, ethyl acetate (EtOAc), and *n*-butanol (BuOH). The *n*-hexane-soluble layer (125 g) was separated into seven fractions (H1–H7) by silica gel (70–230 mesh) column chromatography (CC) eluting with *n*-hexane-EtOAc (1:0 to 0:1, *v*/*v*). Fraction H1 (40 g) was further subjected to silica gel (230-400 mesh) and eluted with *n*-hexane-EtOAc (99:1 to 19:1, *v*/*v*) to afford ten subfractions (H1-1–H1-10). Especially, gomisin L1 (70 mg) was obtained by purifying fractions H1-3 using Sephadex LH-20 CC (CH_2_Cl_2_-MeOH, 1:1 *v*/*v*, 3.6 cm × 72 cm). The purity of gomisin L1 (>95%) was determined by ^1^H NMR analysis (Figure S1). The structure of gomisin L1 was identified by NMR spectroscopic data measurement (Supplementary Figures S1 and S2) and by comparison with published value [14]. Sephadex LH-20 (Amersham Pharmacia Biotech, Buckinghamshire, United Kingdom) and Silica gel (Merck 60A, 230–400 mesh ASTM) were used for column chromatography. All solvents used for extraction, solvent partition, and chromatographic separations were purchased from Ducksan Pure Chemical Co. (Ansan, Gyeonggi-do, Korea) and were distilled before use. Dimethyl sulfoxide (DMSO), propidium iodide (PI), hydrogen peroxide solution (H_2_O_2_), and *N*-acetyl cysteine were purchased from Sigma Chemical (St. Louis, MO, USA). 3-(4,5-Dimethylthiazol-2-yl)-2,5-diphenyl-tetrazolium bromide (MTT) was obtained from Molecular Probes Inc. (Eugene, OR, USA). Dichlorofluorescein diacetate (DCF-DA) was purchased from Santa Cruz Biotechnology (Santa Cruz, CA, USA). Annexin V-fluorescein isothiocyanate (FITC) was procured from BD Biosciences (San Jose, CA, USA).

### 2.2. Cell Culture and MTT Assay

The ovarian cancer cell lines A2780 and SKOV3 were originally obtained from the American Type Culture Collection. RPMI1640, fetal bovine serum (FBS), streptomycin, and penicillin were procured from Life Technologies Inc. (Grand Island, NY, USA). The cells were cultured in RPMI1640 containing 5% FBS, penicillin (100 U/mL), and streptomycin sulfate (100 μg/mL). MTT assay was carried out to measure cytotoxicity. The ovarian cancer cells were plated in a 96-well plate at a seeding density of 0.8 × 10^3^ cells per well and incubated for 24 h. Various concentrations of gomisin L1 (3.12, 6.25, 12.5, 25, 50, and 100 μM) were added to each well and cultured for 48 h. 25 μL of MTT solution (5 mg/mL) was added to each well, and the plates were incubated at 37 °C for an additional 4 h. The medium was discarded, and then 100 μL of DMSO was added into each well to dissolve the formazan blue, which was formed in the cells. The plate was analyzed with a microplate spectrophotometer (Molecular Devices, Sunnyvale, CA, USA) at 540 nm. The IC_50_ value was defined as the concentration at which the number of cells was decreased by 50%, compared to that in the control cultures.

### 2.3. PI Staining for Cell Cycle Analysis

The ovarian cancer cells were plated in a 6-well plate at a seeding density of 0.8 × 10^6^/mL cells per well and incubated for 24 h. The cells were treated with gomisin L1 for the indicated concentrations and times. After washing with cold PBS, the cells were incubated with 70% ice-cold EtOH at 4 °C for 1 h for fixation and permeabilization. The cells were resuspended with PBS and stained with PI solution (50 μg/mL) containing RNase A (5 μg/mL) for 30 min at room temperature in the dark. The cells suspensions (10,000 cells per group) were subjected to the Guava EasyCyte flow cytometers system (EMD Millipore, Darmstadt, Germany).

### 2.4. PI and Annexin V Double Staining

For PI and AnnexinV double staining, on the day of collection, the cells were harvested, washed with cold PBS, and resuspended with 100 mL of binding buffer (10 mM HEPES/NaOH, 140 mM NaCl, 2.5 mM CaCl_2_, pH 7.4). 5 mL of FITC-conjugated AnnexinV and 5 mL of PI (50 mg/mL) were added to the cell suspension and incubated for 15 min at room temperature in a dark place. The cell suspension was analyzed by the Guava EasyCyte flow cytometers system.

### 2.5. Determination of Intracellular Reactive Oxygen Species (ROS)

Fluorescent probe DCFH-DA was subjected to the measurement of the intracellular levels of ROS. The cells were resuspended in PBS and incubated with 20 μM DCFH-DA, and then treated with gomisin L1 at the indicated concentrations and times. The cells were harvested, resuspended with PBS, and subjected to the Guava EasyCyte flow cytometers system.

### 2.6. RNA Interference for Gene Knockdown

The ovarian cancer cells were transfected with small interference RNA (siRNA) using Lipofectamine^®^ RNAiMAX (Invitrogen, Carlsbad, CA, USA) to knockdown the expression of p47^phox^. Control (scrambled) and p47^phox^ and siRNAs (Bioneer technology, Daejon, Korea). The cells were plated in a 6-well plate and incubated to be about 70% confluent at transfection. Lipofectamine^®^ RNAiMAX and siRNAs were diluted with serum-free Opti-MEM medium (Invitrogen, Waltham, CA, USA), separately. The diluted siRNA was added into the Lipofectamine dilute and incubated for 15 min at room temperature. The siRNA-Lipofectamine complexes were gently added to the cells and cultured for 6 h. The transfection mixtures were removed and fresh RPMI1640 medium was added. After 18 h, the cells were used for experimental treatments.

### 2.7. Statistical Analysis

The data are presented as the mean ± SD. One-way analysis of variance and Student’s *t*-test were used to determine statistically significant differences. GraphPad Prism software (GraphPad, San Diego, CA, USA) was used for statistical analyses and graphs. *p*-value < 0.05 was considered to be statistically significant.

## 3. Results

### 3.1. Gomisin L1 from Schisandra Berries Inhibits the Growth of Human Ovarian Cancer Cells

**Figure 1:**
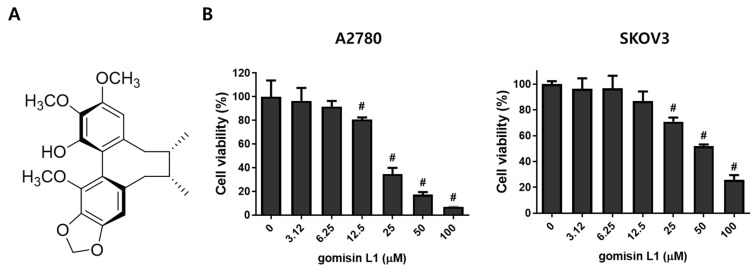
Effect of gomisin L1 on the cell viability of human ovarian cancer cells. (**A**) The structure of gomisin L1 (**B**) The cells were treated with gomisin L1 for the indicated concentrations for 48 h. MTT assay was performed to determine the effect of gomisin L1 on cell viability in human ovarian cancer cells A2780 and SKOV3. *# p* < 0.05 as compared with the control group.

To determine the effects of gomisin L1 (Figure 1A), a lignan isolated from Schisandra berries, on the growth of ovarian cancer cells, the MTT assay was performed in A2780 and SKOV3 human ovarian cancer cells. As depicted in Figure 1B, gomisin L1 significantly inhibited the viability of these cells in a dose-dependent manner. The IC_50_ values (i.e., the concentration that resulted in a 50% decrease in the number of viable cells compared to the control) of A2780 and SKOV3 cells were 21.92 ± 0.73 and 55.05 ± 4.55 μM, respectively. These data indicate that gomisin L1 has inhibitory effects on the growth of human ovarian cancer cells.

### 3.2. Gomisin L1 Induces Apoptotic Death of Human Ovarian Cancer Cells

**Figure 2:**
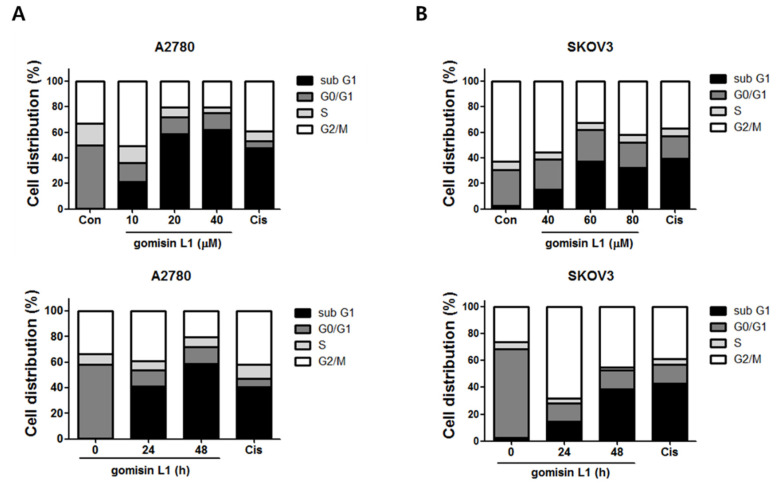
Effect of gomisin L1 on cell cycle regulation in human ovarian cancer cells. (**A**) A2780 cells were treated with gomisin L1 for the indicated concentrations (10, 20, and 40 μM) and times (24 and 48 h). (**B**) SKOV3 cells were treated with gomisin L1 for the indicated concentrations (40, 60, and 80 μM) and times (24 and 48 h). After treatment, the cells were stained with propidium iodide (PI) and P cells cycle distribution profiles of (**A**) A2780 and (**B**) SKOV3 cells were determined by flow cytometry. Representative histograms indicate the distribution of cell population in the cell cycle (sub G1, G0/G1, S, and G2/M phases). Cisplatin, a positive control, was applied at 20 μM.

**Figure 3:**
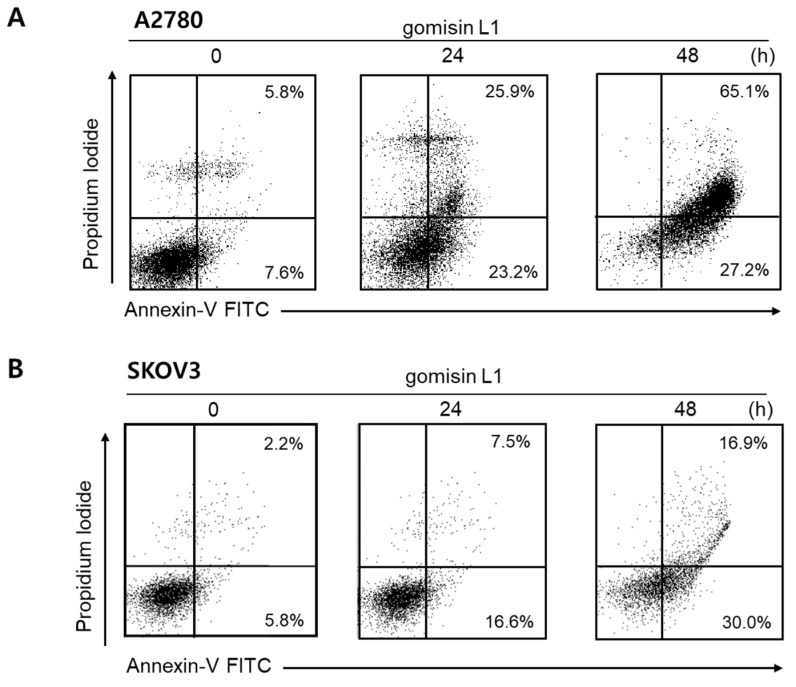
Effect of gomisin L1 on the induction of apoptosis in human ovarian cancer cells. (**A**) A2780 and (**B**) SKOV3 cells were treated with gomisin L1 (20 and 60 μM, respectively) for 24 h and 48 h, and then co-stained with PI and FITC-conjugated Annexin V. The translocation of phosphatidylserine was detected by flow cytometry.

Next, the molecular mechanism underlying the growth-inhibitory effects of gomisin L1 in ovarian cancer cells was investigated. To determine whether these inhibitory effects were related to the induction of cell cycle arrest and/or cell death, the cell-cycle phase distribution of human ovarian cancer cells following treatment with gomisin L1 was analyzed using flow cytometry. As shown in Figure 2, gomisin L1 treatment did not induce cell-cycle arrest, a mechanism of growth inhibition, in A2780 and SKOV3 cells. In contrast, the number of cells in the sub G1 phase (i.e., apoptotic cell death) was increased after treatment with gomisin L1. These data indicate that the gomisin L1-induced growth inhibitory effect was associated with the induction of cell death rather than with cell-cycle arrest. Cisplatin, a platinum drug currently used as a first-line chemotherapeutic agent for ovarian cancer, was used as a positive control. To determine whether gomisin L1-induced cell death is mediated by the induction of apoptosis, the externalization of phosphatidylserine, an apoptotic marker, was analyzed using flow cytometry. A2780 and SKOV3 cells were treated with gomisin L1 and subsequently stained with Annexin V-fluorescein isothiocyanate (FITC) and propidium iodide (PI). As shown in Figure 3, gomisin L1-increased the populations of Annexin V-FITC-positive (apoptotic) cells in the right quadrants of flow cytometry plots in a time-dependent manner. Our findings suggest that gomisin L1-induced inhibition of the growth of human ovarian cancer cells is mediated by apoptotic cell death.

### 3.3. Gomisin L1-Induced Apoptosis Is Associated with Intracellular Reactive Oxygen Species (ROS) Production

**Figure 4:**
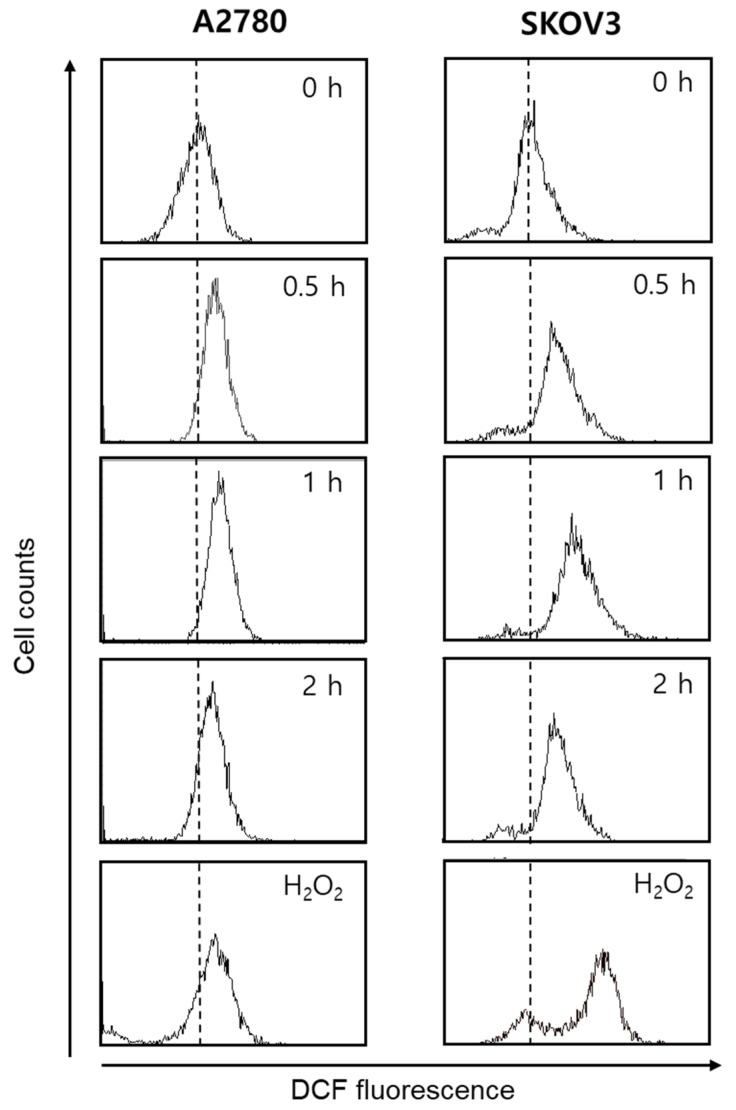
Effect of gomisin L1 on intracellular ROS levels in human ovarian cancer cells. A2780 and SKOV3 cells were treated with gomisin L1 (20 and 60 μM, respectively) for 0, 0.5, 1, or 2 h, stained with DCFH-DA, and then analyzed by flow cytometry. H_2_O_2_, a positive control, was applied at 100 μM.

**Figure 5:**
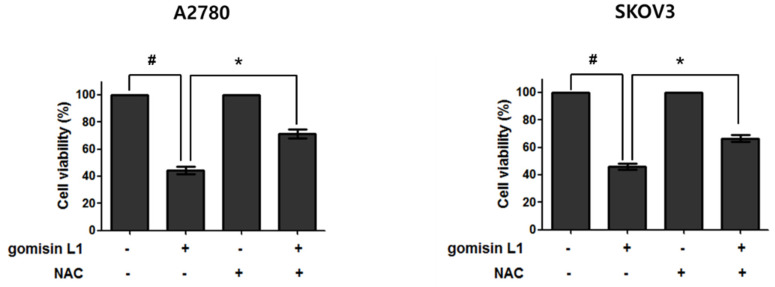
Effect of NAC on gomisin L1-induced cell death in human ovarian cancer cells. A2780 and SKOV3 cells were treated with NAC (5 mM) for 30 min and then treated with gomisin L1 (20 and 60 μM, respectively) for 48 h. MTT assay was carried out to measure the cell viability after gomisin L1 treatment. *# p* < 0.05 as compared with the control group and * p < 0.05 as compared with the gomisin L1-only treated group.

Accumulation of intracellular ROS is an early signal in apoptosis [15,16,17]. Therefore, the involvement of ROS in gomisin L1-mediated cell death was investigated using DCFH-DA. This reagent is cut by esterase and oxidized in the presence of ROS to produce fluorescent 2′, 7′-dichlorofluorescein (DCF). As depicted in Figure 4, treatment with gomisin L1 markedly increased DCF fluorescence in A2780 and SKOV3 cells in a time-dependent manner. The DCF fluorescence intensity increased significantly as early as 30 min after treatment with gomisin L1 (20 μM), showing rapid production of ROS in gomisin L1-treated cells. In addition, gomisin L1-mediated death of A2780 and SKOV3 cells was significantly attenuated by pretreatment with an antioxidant, n-acetyl cysteine (a general free radical scavenger) (Figure 5). These data indicate that intracellular ROS production is involved in gomisin L1-induced apoptosis of human ovarian cancer cells.

### 3.4. NADPH Oxidase Is Involved in Gomisin L1-Induced Cell Death

**Figure 6:**
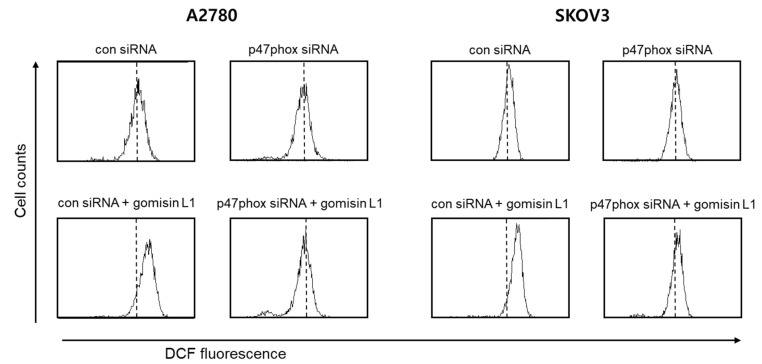
Effect of p47^phox^ knockdown on gomisin L1-induced ROS generation in human ovarian cancer cells. A2780 and SKOV3 cells were transfected with p47^phox^ siRNA or control (Con) siRNA and treated with gomisin L1 (20 and 60 μM, respectively). The cells were stained with DCFH-DA and then analyzed by flow cytometry.

**Figure 7:**
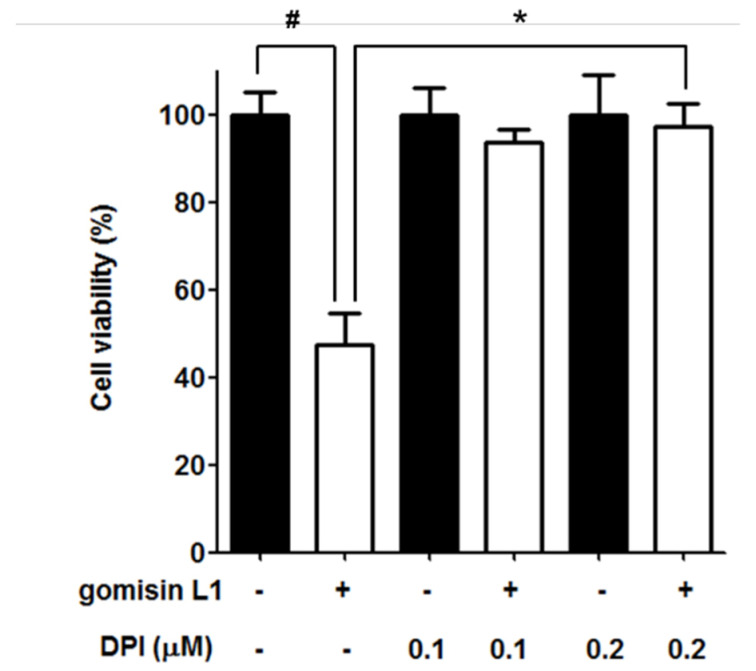
Effect of NOX inhibitor on gomisin L1-induced apoptotic cell death in human ovarian cancer cells. SKOV3 cells were pretreated with NOX inhibitor DPI for 30 min and then treated with gomisin L1 (60 μM). MTT assay was carried out to measure the cell viability after gomisin L1 treatment. # *p* < 0.05 as compared with the control group and * *p* < 0.05 as compared with the gomisin L1-only treated group.

ROS are formed in mitochondria as byproducts of the respiratory chain and are specifically produced by oxidases, such as NADPH oxidase (NOX) [18], which is thought to play crucial roles in cell proliferation and tumorigenesis [19,20]. Therefore, we investigated whether NOX is associated with gomisin L1-induced cell death via ROS production. As shown in Figure 6, knockdown of p47phox of NOX using a specific siRNA suppressed gomisin L1-induced ROS production in A2780 and SKOV3 cells. In addition, the gomisin L1-mediated reduction in cell viability was significantly reversed by the NOX inhibitor, DPI, in SKOV3 ovarian cancer cells (Figure 7). These data suggest that gomisin L1-induced apoptosis of human ovarian cancer cells is mediated by ROS production via NOX.

## 4. Discussion

Schisandra berries are consumed in several Asian countries. The berries contain two major bioactive components: nortriterpenoids and lignans [3,5]. Lignans, formed by the coupling of two coniferyl alcohol residues, have recently been of much interest for their potential health benefits [21]. A recent phytochemical study revealed that the total lignan content of Schisandra berries is ~2000 mg per 100 g dry weight [22], comparable to that of flaxseeds (900–3000 mg per 100 g), one of the richest known food sources of lignans [23]. More than 30 lignans, including schizandrin, gomisin A, gomisin N, deoxyschisandrin, and gomisin L1, have been identified in Schisandra berries [6]. Schisandra berry extracts and their major bioactive lignans have been reported to have various bioactivities (e.g., hepatoprotective, anti-inflammatory, antidiabetic, antioxidant, neuroprotective, and anticancer effects) [1,4,8,9,10]. However, most studies have focused on their hepatoprotective and anti-inflammatory activities, and a few have addressed their anticancer activities and underlying molecular mechanisms. For example, *Schisandra chinensis* fruit extract induced apoptosis via p38/JNK activation and the ROS-mediated/mitochondria-dependent pathway in human gastric cancer cells [24]. Gomisin A-induced G1 cell-cycle arrest in HeLa cervical cancer cells [10] and apoptosis in HCT116 colon cancer cells [25]. Schizandrin inhibited cell proliferation by inducing cell-cycle arrest in T47D breast cancer cells [26]. Gomisin N enhanced TRAIL-induced apoptosis by upregulating death receptors 4 and 5 in HeLa cells [27]. Schisandrin B and shchizantherin C inhibited the growth of A549 lung cancer cells by inducing cell-cycle arrest or apoptosis [7]. In addition, schisandrol A, schizandrin A, schizandrin B, and schisantherin A reversed p-glycoprotein-mediated multidrug resistance [28,29]. In a mouse model, gomisin A inhibited 12-*O*-tetradecanoylphorbol-13-acetate (TPA)-induced carcinogenesis. In a previous study, we also demonstrated that deoxyschizandrin, a major Schisandra lignan, induced cell cycle arrest in ovarian cancer cells and inhibited the protumoral activation of tumor-associated macrophages.

Although there are many reports on the pharmacological activities of other lignans from Schisandra berries, only a few studies on gomisin L1 were reported [13,30]. A study reported that gomisin L1 showed mild cytotoxicity against HL-60 leukemia and HeLa cervical cancer (IC_50_ = 82.02 and 166.19 μM, respectively), and no cytotoxicity MCF7 breast cancer cells (IC_50_ > 200 μM) [30]. Thus, in this study, the antitumor effects of gomisin L1 on human ovarian cancer cells were evaluated. Gomisin L1 significantly inhibited the growth of A2780 and SKOV3 human ovarian cancer cells. Moreover, the gomisin L1-induced inhibition of growth was mediated by apoptotic cell death rather than cell-cycle arrest. These findings suggest that gomisin L1 has potential as an anticancer agent, and this is the first report of the biological activity of gomisin L1. Apoptosis can be induced by the extrinsic pathway, which is also known as the death receptor pathway, and the mitochondrial-dependent intrinsic pathway [31]. In the death receptor pathway, death receptors such as tumor necrosis factor-α or Fas receptors are stimulated by specific death ligands. In contrast, the intrinsic pathway is stimulated by a variety of extracellular and intracellular death stimuli, such as ROS and DNA damage. Whether gomisin L1 induces apoptosis through the extrinsic or intrinsic pathway or both pathways remain to be further investigated.

ROS are involved in the regulation of various cellular responses, such as proliferation, differentiation, angiogenesis, gene expression, immune regulation, and apoptosis. High levels of intracellular ROS induce cell-cycle arrest, inhibit proliferation, and stimulate apoptosis, resulting in inhibition of tumor cell growth [32,33,34]. ROS are thought to play a key role in apoptotic cell death through both the intrinsic and extrinsic pathways [35,36,37]. For example, ROS activate the intrinsic pathway by modulating mitochondrial outer membrane permeability, releasing cytochrome c from the mitochondria to the cytosol, and stimulating apoptotic caspase cascades [35]. In addition, ROS production stimulates the assembly of death receptors and activation of FADD-caspase-8 cascade and apoptosis signal-regulating kinase 1, an extrinsic apoptotic signal [37,38]. In fact, ROS accumulation is related to the apoptotic response to several chemotherapeutic reagents, including platinum-based chemotherapeutics such as cisplatin, which is the most effective drug for treating ovarian cancer [36,38]. In addition, many natural compounds have been reported to induce ROS-dependent apoptosis of cancer cells through both the extrinsic and intrinsic pathways [39,40]. The findings of this study demonstrate that treatment with gomisin L1 induces apoptosis in human ovarian cancer cells by enhancing ROS generation. Many natural polyphenols, including Schisandra lignans, have antioxidant activities and cytoprotective effects by reducing ROS levels [41,42,43,44]; however, there have been no reports on the antioxidant activity of gomisin L1. Interestingly, several lignans including Schisandra lignans that are known to have antioxidant activity, have been demonstrated to induce ROS production, resulting in the apoptosis of cancer cells [45]. For example, sesamol, a major lignan in sesame seeds, induced apoptosis by ROS production and JNK-dependent mitochondrial damage in HCT116 colon cancer cells [46]. Moreover, schisandrin B, gomisin J, and gomisin N, lignans isolated from Schisandra barriers, also have shown to inhibit the cancer cells’ growth by inducing apoptosis via ROS production [27,47,48].

The mechanism by which gomisin L1 induces ROS production in ovarian cancer cells was also evaluated in this study. ROS are mainly formed in mitochondria as byproducts of the respiratory chain. In addition, ROS are specifically produced by oxidases, such as arachidonic acid, oxygenase xanthine oxidase, and NOX [18]. NOX is expressed in various cancer cells including ovarian cancer cells [49,50]. NOX is a transmembrane protein that produces ROS via the oxidation of NADPH [51], and interestingly, plays a crucial role in the proliferation and death of cancer cells [52]. For example, NOX-mediated redox modulation was associated with the tolerance of cancer cells to anticancer drugs [52]. Upregulation of NOX led to TGF-induced apoptosis in hepatic cancer cells [53]. Additionally, cisplatin-induced death of human prostate cancer cells via NOX-dependent ROS production [54]. As NOX is involved in ROS-dependent apoptosis of cancer cells, whether gomisin L1-induced production of ROS was associated with NOX in human ovarian cancer cells was investigated. The siRNA knockdown of p47^phox^, a key protein in the assembly of NOX [55], significantly suppressed gomisin L1-induced ROS production; treatment with the NOX inhibitor DPI significantly inhibited the gomisin L1-induced death of ovarian cancer cells. These data suggest that gomisin L1-induced apoptosis is mediated by NOX-dependent ROS production.

This is the first study to demonstrate that gomisin L1 triggers apoptotic death of human ovarian cancer cells via ROS production through NOX. These findings expand our understanding of the health benefits and possible nutraceutical applications of gomisin L1.

## Supplementary Materials

The following are available online at https://www.mdpi.com/article/10.3390/life11080858/s1, Figure S1: 1H-NMR spectrum of gomisin L1 from the fruits of Schisandra chinensis (400 MHz, CDCl3), Figure S2: 13C-NMR spectrum of gomisin L1 from the fruits of Schisandra chinensis (100 MHz, CDCl3).
