# Supplementary material for: Gomisin L1, a Lignan Isolated from Schisandra Berries, Induces Apoptosis by Regulating NADPH Oxidase in Human Ovarian Cancer Cells
Source: Life (Basel). 2021 Aug 21;11(8):858. doi: 10.3390/life11080858 (PMC8398161; doi:10.3390/life11080858)
Supplement: Supplementary file 1 [file life-11-00858-s001.zip › life-1345969-supplementary.pdf]

life

Supplementary Materials

Gomisin L1, a Lignan Isolated from Schisandra Berries, Induces Apoptosis by Regulating NADPH Oxidase in Human Ovarian Cancer Cells

Young-Hyun Ko [1], Miran Jeong [1], Dae-Sik Jang [2], and Jung-Hye Choi [1,2,*]

[1] Division of Molecular Biology, College of Pharmacy, Kyung Hee University, Seoul 02447, Korea; kyh122@khu.ac.kr (Y.H.K.); jeongmr@khu.ac.kr (M.J.)
[2] Department of Biomedical and Pharmaceutical Sciences, Kyung Hee University, Seoul 02447, Korea; dsjang@khu.ac.kr
* Correspondence: jchoi@khu.ac.kr

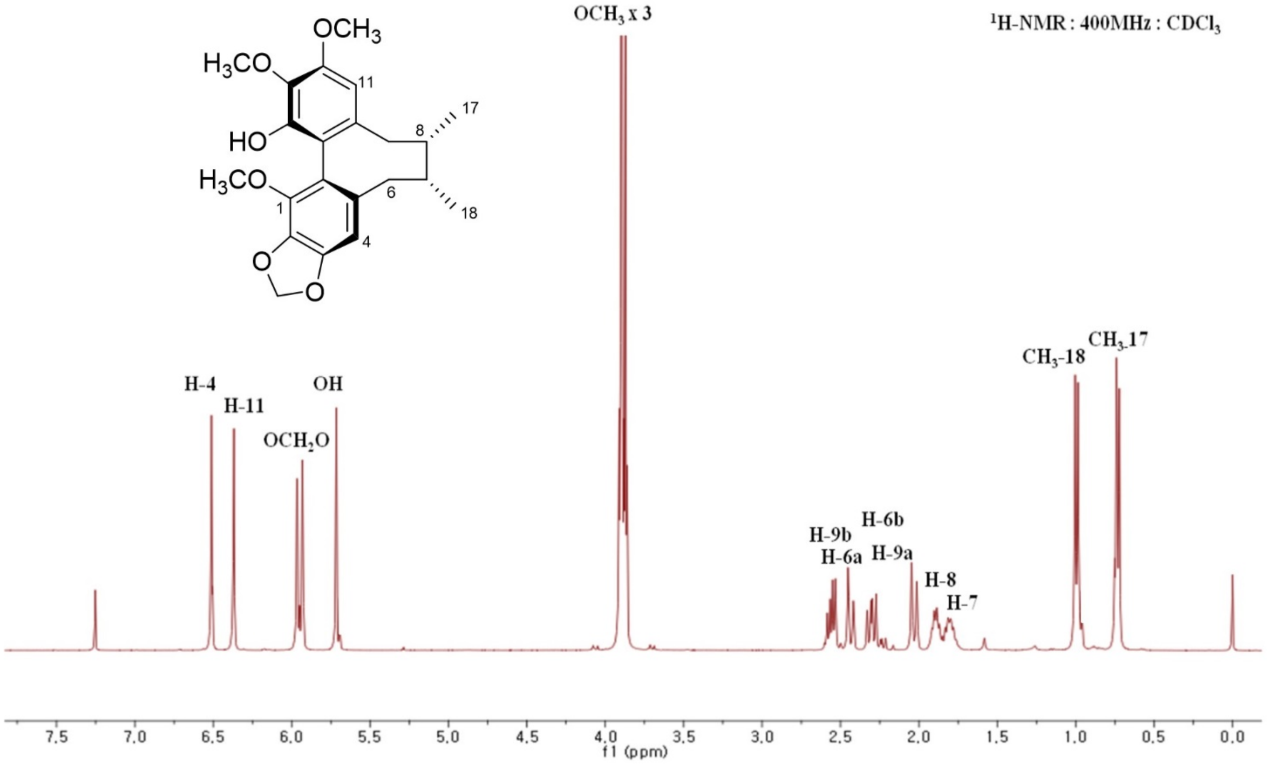

Figure S1. ^{1}H-NMR spectrum of gomisin L1 from the fruits of *Schisandra chinensis* (400 MHz, $CDCl_3$).

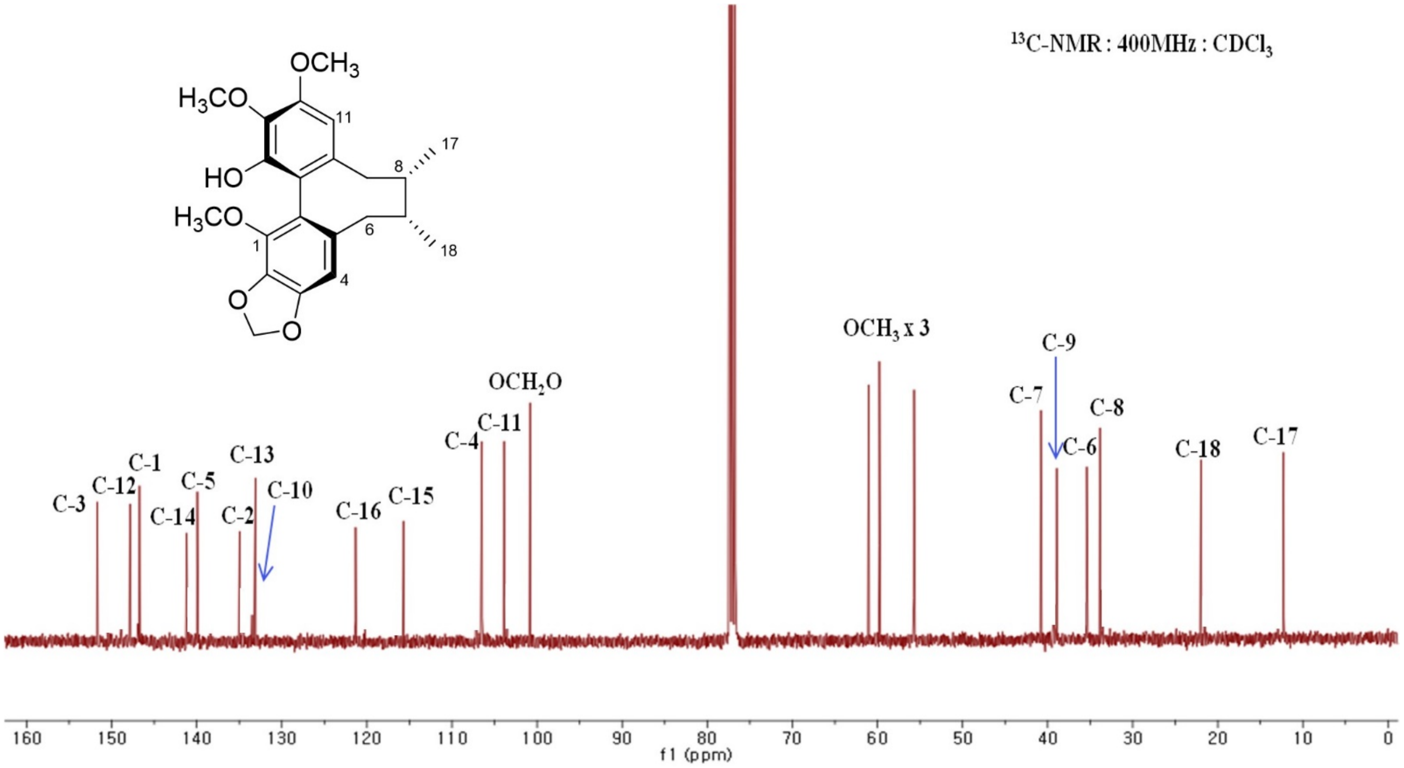

Figure S2. ^{13}C-NMR spectrum of gomisin L1 from the fruits of *Schisandra chinensis* (100 MHz, $CDCl_3$).
